# “My body is a cage”: A qualitative investigation into the self-discrepancy experiences of young women with metastatic cancer

**DOI:** 10.1177/17423953231168014

**Published:** 2023-04-10

**Authors:** Ozlem Kahraman-Erkus, Yagmur Ar-Karci, Tülin Gençöz

**Affiliations:** 1 Department of Psychology, Başkent University, Ankara, Turkey; 2 Department of Psychology, TED University, Ankara, Turkey; 3 Department of Psychology, Middle East Technical University, Ankara, Turkey

**Keywords:** Self-discrepancy, self-concept, metastatic cancer, young adulthood, chronic disease

## Abstract

**Objectives:**

The current study investigated self-discrepancy experiences of young women with metastatic cancer.

**Methods:**

Semistructured interviews were conducted. Data were analyzed through interpretative phenomenological analysis.

**Findings:**

Eight female patients with metastatic cancer aged between 27 and 38 years formed the sample. Three superordinate themes emerged: (1) compulsory changes in self-concept with ambivalent evaluations; (2) new ideals not on the agenda of a healthy young woman; and (3) so-called ‘minimalist’ expectations from others.

**Discussion:**

Findings indicated that diagnosis and treatment of metastatic cancer impose unique developmental challenges for young adult women. Advanced cancer disrupted the tasks and responsibilities of young adulthood, resulting in frustration, grief, isolation, and overcompensation. These findings suggest that a developmental perspective is crucial when working with self-discrepancy experiences of young women with metastatic cancer.

## Metastatic cancer

Cancer is one of the most life-threatening diseases,^1,2^ and the stage of the disease has a pivotal role in determining the prognosis and treatment outcomes. Particularly, metastatic cancer is associated with increased mortality,^3,4^ treatment failures,^5^ psychological symptoms,^6–8^ perceived symptom severity,^9–11^ and caregiver burden.^12^ Although various psychological impacts might be evident across different age groups (e.g., social isolation, sleep disturbance, depression, and anxiety), young adults with cancer seem to experience particular challenges due to their unique developmental conflicts and responsibilities.^13^ The reason is advanced cancer disrupts such developmental tasks as assuming one's own responsibilities, gaining social/economic independence, and juggling multiple roles within and outside of the family system.^14–18^ Metastatic cancer interferes with autonomous functioning and patients typically need profound assistance and help during treatment.^19,20^ Young patients feel particularly distressed because of the physical and psychosocial losses associated with a potentially fatal prognosis.^21,22^ For young adulthood it is a period in which individuals navigate their identity through intimacy and career development, autonomous decision making, and psychological differentiation.^23,24^ It is, therefore, important to examine how being diagnosed with metastatic cancer interferes with the self-concept and self-discrepancy experiences of young adults.

## Self-discrepancy and cancer diagnosis

**Figure 1.**
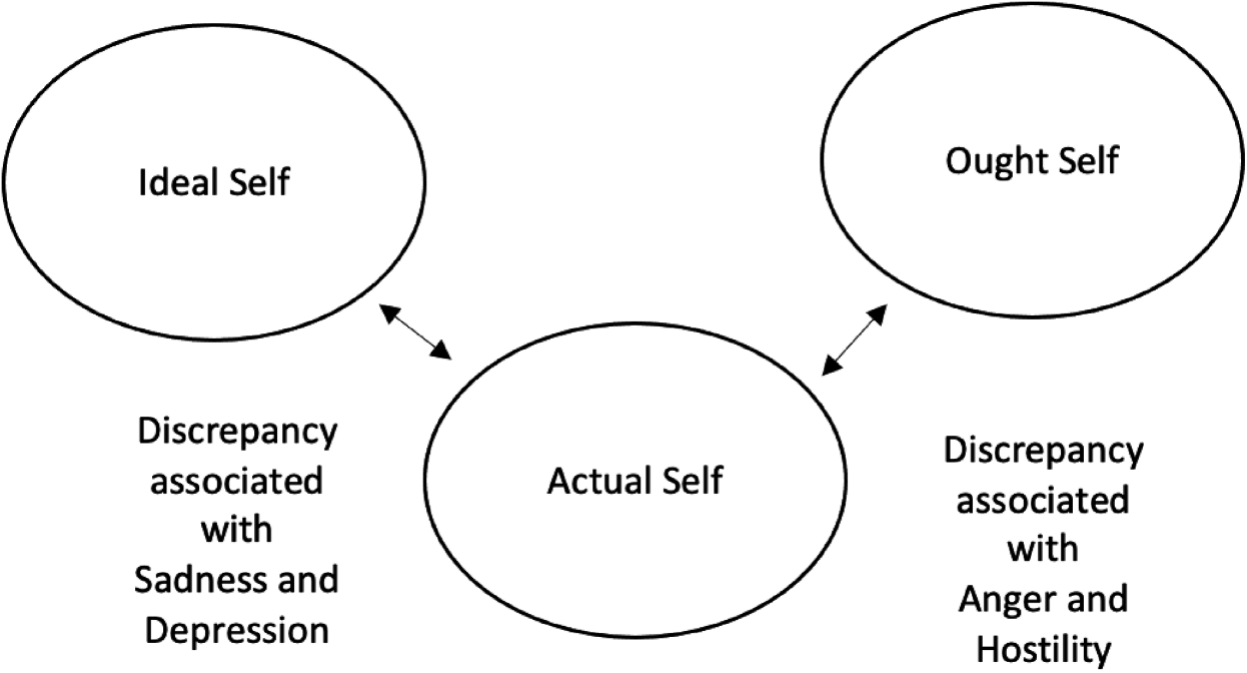
Self-discrepancy theory.

Cancer inevitably causes changes in perceptions of self, others, and the world.^25,26^ Young adults with metastatic cancer may experience a profound shift in their self-concepts as their health status is incompatible with their life plans. Higgins is the first who referred to such conflicts among different domains of self through his Self-Discrepancy Theory.^27^ Self-domains consist of actual (i.e., characteristics one thinks they own), ideal (i.e., characteristics one desires or hopes to own), and ought (i.e., attributes one feels obliged to attain) selves. These domains are grounded on the perceptions of both one's self and others. Self-discrepancy occurs when there is an incongruence between the actual self and the other two domains (i.e., ideal and ought), and it is associated with emotional vulnerabilities such as dejection, depression, and agitation.^27^ While should own self-discrepancy (i.e., the discrepancy between the actual self from one's own point and the characteristics one thinks they should oblige to possess) has been associated with anger and hostility, ideal own self-discrepancy (i.e., the discrepancy between the actual self and the characteristics one desires or hopes to possess from one's own point of view) has been found to be more closely related with sadness and depression^28,29^ (see Figure 1).

Self-concept is a broad term conceptualized as self-evaluative judgments about one's attributes, abilities, self-worth, and potential.^30^ Self-concepts of patients with cancer have been exclusively investigated in relation to body image, self-esteem, self-efficacy, or general self-perception^31–33^ without focusing on the impacts of self and other oriented beliefs. To the authors’ knowledge, only two studies have so far examined the self-discrepancy experiences of patients with cancer. According to a quantitative study conducted with elderly women, patients with cancer were found to have lower ideal self scores compared to women having other health problems. The patients with cancer kept their self-discrepancy at an optimal level by lowering their expectations of the ideal self.^34^ Another study concluded that higher levels of self-discrepancy are associated with greater depression, lower psychological health, and a weakened purpose of life regardless of the severity of the symptoms.^35^ However, the ages of both sample groups were 60 on average, and self-discrepancy experiences have not yet been studied for young adult patients with cancer. Furthermore, since the symptom burden of metastatic cancer is higher,^9^ these effects should be specifically examined in patients diagnosed with metastatic cancer.

As there is a close relationship between self-discrepancy and psychological health,^36,37^ investigating the impacts of metastatic cancer on different domains of self-concept is essential. Being diagnosed with metastatic cancer is challenging for all patients, but its effects may vary depending on the patient's developmental period. Particularly, young adulthood might be a vulnerable period of life. Obstacles to autonomy, individuation, and independence, the main developmental tasks of young adulthood,^38,39^ might lead to changes in different aspects of self-concept. Thus, this group needs special examination to obtain in-depth knowledge of self-discrepancy experiences pertaining to metastatic cancer. The age range of this study was determined according to Erikson's description of the young adulthood stage.^40^ This age period was particularly preferred because it is also compatible with the reports of the National Cancer Institute^41^ while detailing facts about types of cancer in young people. The research question was formed as follows:

1. “What are the self-discrepancy experiences of young adults with metastatic cancer?”

## Method

In the following sections, the methodological information of the research is presented. The consolidated Criteria for Reporting Qualitative Studies (COREQ)^42^ checklist is used.

### Research team and reflexivity

All the interviews were conducted by the first author (OK-E) from December 2018 to June 2019. The first researcher was working as a clinical psychologist at the Oncology Department of Hacettepe University Hospital and doing her PhD at the time of data collection. The other researchers have PhD degrees in clinical psychology and have expertise in qualitative research. No prior relationship existed between investigators and participants. All participants were informed about the purpose of the study and audio recording. Since the two researchers (OK-E and YA-K) and the participants were in the same developmental period, it was thought that there might be a potential bias in the interpretation of the data. To minimize it, conceptual feedback was obtained from the third researcher (TG) who was a middle-aged woman working as a clinical psychology professor at Middle East Technical University.

## Study design

### Theoretical framework

Interpretative phenomenological analysis (IPA) provides an extensive understanding of how individuals experience a specific phenomenon, and how they make sense of it.^43^ IPA is particularly suggested for less commonly researched topics to provide an initial framework about the subject of interest. To the authors’ knowledge, studies on the self-discrepancy experiences of patients with cancer are limited, and there is no study focusing particularly on the self-discrepancy experiences of young adults with metastatic cancer. For this purpose, IPA was chosen to provide a thick description of the discrepancies among different domains of self through investigating how young adults make sense of metastatic cancer.^44^

### Participant selection

IPA requires purposive sampling to obtain a homogeneous sample in terms of the subject of interest.^44^ Although the preliminary focus of the study was to investigate the self-discrepancy experiences of young adults with metastatic cancer, the inclusion criterion was narrowed down based on the participants’ gender. Being a female was added as another criterion to ensure homogeneity since only female participants responded to the first announcements of the study. After completing the interviews with three young women, the inclusion criteria were revised as follows: (1) being a young adult between the ages of 20 and 39 years, (2) undergoing active treatment at the time of the study, (3) being aware of the current diagnosis, and (4) being a female patient with metastatic cancer. To recruit potential participants, a convenience sampling method was used. Nine participants meeting the criteria were invited to the study, but one of them did not want to participate for personal reasons. In consistent with IPA guidelines,^44^ the data collection process was terminated after the eighth interview since data started to repeat itself and obtained themes reached conceptual saturation.

### Setting and data collection

Face-to-face semistructured interviews were conducted. All interviews were conducted in a room where the participant and the researcher were alone at the Hacettepe University Hospital. Only one interview was held with each participant and the mean duration of the interviews was 55 min. Interview questions were predefined, open-ended, and far from directions (see Appendix 1). Questions were developed by the research team to examine the changes in the different dimensions of self-concepts after being diagnosed with metastatic cancer. A pilot interview was conducted to test the suitability of the questions in terms of content and structure. A research team examined the transcript of the pilot interview and no changes had been made to the initial question list as the data related to the subject topic was sufficiently probed through those questions. Each interview started with warm-up questions eliciting information about the metastatic disease process (e.g., Could you tell me about your cancer diagnosis process? How did you learn the metastatic diagnosis? How did you feel when you learned the current diagnosis?). Subsequent questions were designed to obtain information about current self-perceptions, ideal attributes, and obligations. Finally, participants were asked how they felt about participating in this study. All the participants stated that they were not disturbed by the interview questions.

### Analysis

In analyzing the data, an inductive perspective was employed, consistent with the rationale of IPA.^45,46^ As recommended, analyses of the participants’ accounts were performed case by case following an iterative process.^44^ The first step was to create a word-for-word transcript from the audio recording of the first interview. The transcription process was performed by the first author (OK-E). The transcript of the first interview was then coded according to its content, language, and interactional context by the first and second researcher independently (OKE and YAK). Short notes taken during and after the interview were also used in the coding stage. The codes were grouped consistently with their conceptual relations. In this way, a list of subordinate and superordinate themes was determined separately by both researchers. The findings of the analysis for the first case were shared with the research team (OKE, YAK, and TG) in weekly meetings, and conceptual discussions were held until a consensus was reached. Then, the same analytic process was employed for the second interview. A cross-case comparison was performed between the theme lists of the first and second interviews, and a common theme list was developed in weekly meetings. The same process was repeated exactly for the analysis of all remaining cases one by one. After each analysis, a cross-case comparison was made with the last common theme list. Previous analyses were re-examined for new themes emerging in the following interview.

The self-discrepancy theory was used as an initial framework when forming the research question and interview questions. As presented in the setting and data collection sections, questions were formed to elaborate on how metastatic disease affected different dimensions of self-concept during young adulthood. Consistent with the IPA's guidelines,^44^ no explicit question was asked referring to the incompatibility among different dimensions of the self. Instead, young women were allowed to describe their subjective experiences about the impacts of metastatic cancer on different domains of the self and report any incongruences. During data analysis, researchers remained flexible to uncover unexpected themes as well as inspecting shifts and contradictions in the self-concept.

In the following section, all superordinate themes, representing all participants’ nuanced experiences, were described with the most representative quotations. Pseudonyms were used to assure confidentiality.

## Findings

In this study, the following superordinate themes emerged: (1) compulsory changes in self-concept with ambivalent evaluations; (2) new ideals not on the agenda of a healthy young woman; and (3) so-called minimalist expectations from others (see Table 1).

**Table 1.** Themes of interpretative phenomenological analysis of young adult female patients with metastatic cancer in terms of self-discrepancy.

1. Compulsory changes in self-concept with ambivalent evaluations 1.a. Overvaluation of being strong: pushing the limits 1.b. Justification of the characteristics of self after cancer 1.c. Ambivalent emotions about cancer-related losses: laughing while losing
2. New ideals not on the agenda of a healthy young woman 2.a. Normalcy as the new ideal 2.b. Need for an omnipotent self to beat cancer
3. So-called “minimalist” expectations from others 3.a. Expectations centered only around survival 3.b. Restrictions due to others’ pressure about staying strong 3.c. Feeling guilty about the burden imposed on the family by cancer

### Participants

The current sample comprised 8 female patients with metastatic cancer aged between 27 and 38 years. These participants were undergoing active treatment at Y University Oncology Hospital. Although one patient was not diagnosed with metastatic cancer, she was included in the study as her disease, glioblastoma, affects more than one area of her body and was regarded as metastatic. Only female participants were included in the current sample for both practical and theoretical reasons. At the time of the initial announcements about the study and participant criteria, only female patients responded. Thereafter, males were excluded from targeted participation to assure sample homogeneity since gender roles might impose differential dynamics for the developmental tasks of young adulthood.^47^

Demographic and diagnostic information of the participants are presented in Table 2. The primary diagnoses of the participants are given and the areas with metastases are indicated in brackets.

**Table 2.** Demographic and diagnostic information of participants.

Name	Age	Marital status	Education	Treatment	Diagnosis	Time since diagnosis
Asya	27	Single	University	Chemotherapy	Throat (air Tube & lung & liver)	6 years, 11 months
Derin	33	Single	University	Chemotherapy	Colon (lung & liver)	9 months
Fatma	38	Single	University	Chemotherapy	Breast (lung & liver)	5 years, 2 months
Emel	27	Single	University	Chemotherapy	Glioblastoma	4 years, 5 months
Seda	27	Single	University	Chemotherapy	Soft tissue (lung & uterus)	2 years, 1 months
Canan	30	Single	University	Hormone Therapy	Adenoid cystic (lung & bone)	9 years 10 months
Oya	36	Single	University	Chemotherapy	Stomach (ovary)	7 months
Ayşe	37	Single	University	Chemotherapy	Soft tissue (lung)	1 year

### Compulsory changes in self-concept with ambivalent evaluations

This superordinate theme covers the effects of living with metastatic cancer as a young woman and its impact on self-perception. While participants emphasized the strength of their positive emotions, they paradoxically expressed feelings of weakness, loss, and grief. In summary, this superordinate theme reflected the complex effects of “struggling” with the diagnosis of metastatic cancer as a young woman and its impact on self-evaluations.

#### Overvaluation of being strong: Pushing the limits

This subtheme represents the “extreme efforts” participants made to remain as healthy as their peers. They tried to continue their lives the same way before the cancer diagnosis. This theme also covered acting “as if” there had been no physical or psychological losses due to metastatic cancer. Indeed, participants believed that such an attitude made them “different” from older patients. Interestingly, they expressed joy over bystanders’ admiration and surprise in response to their physical and psychological stamina. Still, pushing physical limits to remain “young and healthy” seems to have its consequences. The participants usually dismissed their physical needs and ignored doctors’ recommendations. For example, Asya (age 27, throat cancer) felt proud for continuing work despite her doctors’ recommendations:

“They say ‘strong,’ I heard them saying it a lot. They say things like ‘You are so strong—We- I couldn’t behave like that if I were in your shoes’ … I couldn’t breathe easily, and I couldn’t walk, but I continued working with those physical limitations for 4 months. For example, my colleagues were not aware of my disease. When I talked about the disease, they were very impressed by my efforts. ‘How can you do it,’ they asked—there was no such thing in my thoughts, such as ‘Why did it happen to me?’ or something like that—I am a very positive person.”

#### Justification of the characteristics of self after cancer

This theme is about our patients’ overvaluation of the positive self-related changes brought about by metastatic cancer. Throughout the interviews, almost all participants repeatedly underlined how much they improved “thanks to cancer.” After diagnosed with cancer, they learned to “stay in the moment,” “not mind trivial matters,” and appreciate life more. However, the researchers observed that some participants perceived every personal change as “positive” without sufficient elaboration on their emotions. This observation suggests that participants over-emphasized the silver lining as a compensation strategy. For example, Seda (age 27, soft tissue cancer) reported feeling grateful for having cancer:

“Actually, it was good for me to completely change as a person because I was a very negative person before cancer. I was questioning why I am living this life, now I turn out to be someone who says, ‘I have to live!’ So, I think it's good that I have changed… I hesitate to say it, but thank God I have cancer, it's so good to have cancer, yes, it's hard, but I think it's great. Otherwise, without cancer, I wouldn’t be the person I am now.”

#### Ambivalent emotions about cancer-related losses: Laughing while losing

This theme was about the expression of conflicting emotions while mentioning cancer-related losses. The participants acknowledged that they lost much due to the metastatic cancer diagnosis. They felt extreme weakness and suffered many physical ailments because of chemotherapy. However, almost all of them mentioned those losses with laughter. They even made jokes and mocked themselves while talking about the physical impairments. Some participants cried and laughed simultaneously, indicating conflicting emotions about the profound losses they experienced at such a young age. This was interpreted as a reflection of the ambivalence resulting from having a metastatic disease at a young age. For example, Derin (age 33, colon cancer) explained with laughter that she could not walk without someone's support during the treatment process and that she fell one day while trying to move alone:

“After chemotherapy, when I was at the hospital first - I even fell after chemotherapy, I mean, I tried to move when I got out of the shower, and I was stuck on the ground [laughing]. Because my body was very weak, I lost a lot of weight, my head was constantly spinning, and so my legs were weak. I couldn’t even walk without any support. Someone held my arm all the time.”

### New ideals not on the agenda of a healthy young woman

This superordinate theme depicts the changes in the ideals, dreams, and wishes of young patients with cancer. Young adulthood is a period of dynamic developmental changes. However, there seems to be a significant transformation in the ideals and dreams of young adults with metastatic cancer.

#### Normalcy as the new ideal

This theme is about how the ideals and priorities of female young adult patients with cancer changed and “being normal” became an ideal after a metastatic cancer diagnosis. Our participants had gone through profound changes in their lives. While normalcy was taken for granted before cancer, it became an ideal to be attained throughout the treatment process. Almost all participants stated that their desire to be healthy had overridden all other dreams and wishes. Particularly, they longed for normalcy, ordinary life, and daily hassles. For example, Emel (age 27, glioblastoma) felt she fell behind in life due to her illness. When asked about her wishes and desires, she said performing the ordinary tasks of daily life was her biggest dream:

“[I wish to be] healthy. And I miss being a part of life. I miss dressing as I want, not being forced to only one type. Now I must wear whatever is comfortable because my mobility is limited. Also, since I have received chemotherapy, there should be no bleeding in my body; for example, I can’t wear anything tight. I want to wear whatever I want, like before. I miss doing my makeup. Because I am a person who really cares about her appearance.

#### Need for an omnipotent self to beat cancer

This theme represents the desires of young women for self-transformation. They believed that they had to make necessary changes in their personality, feelings, and thoughts to achieve remission. Their old personality characteristics were held responsible for the development and recurrence of cancer. That is why they made great efforts to develop an ideal personality full of positive attributes putting cancer at bay. However, the development of these new ideal traits seems to have been temporary since they were abandoned whenever participants entered remission. For example, Fatma (age 38, breast cancer) expressed her regret by stating that “I had survived through a transformative experience, I tried to be completely different [after the first remission], but when I started to feel concerned for ordinary life hassles, my cancer had metastasized to my lungs.” Similarly, Emel (age 27, glioblastoma) thought she would be able to overcome the disease by establishing a strong sense of control. She also stated that she would become healthier by dealing with negative events through positive thoughts:

“Previously, I was always negative. For example, if any part of my body hurt, I would believe it was due to something sinister. I try to be positive now. For example, if I have a headache, I interpret it as a sign of healing. Also, when I hear something in my ears, I consider it as a sound of getting better, or tumors disappearing. For example, my thumb nail falls off. When I was first diagnosed, something like that had happened to my nail then. I said, ‘It started like this; it will completely leave my body the same way it started.’ So, it is just healing… I do believe that.”

### So-called minimalist expectations from others

This superordinate theme reflects the duties and responsibilities felt by the participants after the metastatic cancer diagnosis. Participants felt the pressure that they did not have any responsibilities and duties other than surviving cancer. In other words, although the number of expectations seems to decrease, one important and overwhelming task was still present: managing cancer, not crying, and staying alive. Additionally, participants thought the life of others (e.g., parents and family members) around them also became much more difficult because of their illness. This thought leads them to feel profound guilt which seems to accentuate the impacts of others’ expectations of being strong and healthy.

#### Expectations centered only on survival

This theme represents the perception that “nothing is expected of me apart from becoming healthy.” Participants felt the heavy burden of trying to achieve full remission since recovery was not completely under their control. Although cancer seems to free them of any ordinary responsibilities, their one and only duty of staying alive and healthy was the most difficult one. In fact, this might be why defeating cancer was described not only as a desire but also as a responsibility by the participants. For example, Derin (age 33, colon cancer) stated that she feels “like I must recover like it is simply my responsibility towards everyone… I feel that my biggest responsibility is to recover right now.” Not surprisingly, not only the people around them but also the participants themselves expected themselves to defeat cancer. Canan (age 30, adenoid cystic) responded very quickly when asked what she expected of herself, stating: “So, of course, healing [while laughing].”

#### Restrictions due to others’ pressure about staying strong

This theme is particularly about the expectations of others (e.g., family members, doctors, and friends) not to be sad and not to cry during cancer treatment. Participants seem to comprehend an implicit message behind their families’ supporting comments. This implicit message is that the cancer diagnosis can only be beaten by staying strong and positive. This expectation placed burden upon participants and made them reluctant to share their real thoughts and feelings as the expression of negative emotions was associated with “giving up the fight.” Participants also conveyed that their feelings related to metastatic cancer were invalidated and unappreciated through such comments. The following excerpt exemplifies Ayşe's (age 37, soft tissue cancer) anger aroused in response to the assigned duty of remaining positive:

Ayşe: “They want me to be always positive.”

Interviewer: “How does this make you feel, everyone having such an expectation?”

Ayşe: “It is like a burden on my shoulders, because, I mean…they are not in my shoes, I mean, would they be able to remain positive if they were in my place?”

#### Feeling guilty about the burden placed on the family

This theme is related to the feelings of guilt felt by our participants as their disease was not only a burden upon them but upon their loved ones. They acknowledged that their families’ lives became more difficult due to cancer-related problems making them experience profound guilt. Unfortunately, this guilt seems to be another reason for their compulsory happiness. For example, Emel (age 27, glioblastoma) thought that she had “worn out her family.” To compensate for the guilt she experienced, she disguised how she actually felt:

Emel: “In this process, as I have mentioned earlier, I have worn my family out too much, beyond my control.”

Interviewer: “Beyond your control, exactly.”

Emel: “My family is also aware of [the feelings of guilt]. They even get angry with me. I mean, they get angry because I feel guilty. That is why I want to have quality time when I get better. For now, for example, if I get up and dance when I have a headache, I do it to make [the family] happy, to make them smile, make a joke, even if I feel quite ill. I want our lives to be more beautiful than I can imagine when I am recovered.”

## Discussion

The present study investigated the dimensions of self-concept and self-discrepancy experiences of young adult women with metastatic cancer. The first superordinate theme, “compulsory changes in self-concept with ambivalent evaluations,” refers to how the metastatic cancer process affected the self-conceptualizations of young women through impairment in several domains of life. All subthemes together suggested that interviewees pushed their physical and psychological limits to function like young women without cancer. Although they were aware of their cancer-related losses, their efforts to compensate for the impacts of the disease were evident throughout the interviews. Although the focus on having the emotional and physical stamina to cope with cancer-related changes is not something new in the psycho-oncology literature,^48,49^ younger women seem to be torn between the need to stay strong and to grieve for their cancer-related losses. As they reported, “cancer is like a cage” preventing their bodies and souls from functioning at their fullest. In accordance with the present findings, previous research demonstrated that chronic life-limiting illnesses (e.g., multiple sclerosis^50,51^ and HIV^52,53^) are associated with debilitating impacts on family functioning, finances, working life, and daily routines among working-age adults^54^ (i.e., patients aged between 25 and 60 years). Similarly, working-age patients with advanced cancer reported frustration for losing their independence and expressed their need for extensive assistance due to physical symptoms and treatment side effects.^54–56^ This increased dependence made them feel like a burden to family members, further increasing their emotional distress.^57^ Younger adults with cancer face the possibility of impending death when they are expected to manage several responsibilities in the family, community, and professional areas.^54^ The physical limitations and burden of treatment inevitably impede the central plans of young adulthood, like establishing a family, having children, and advancing professionally.^58–60^ Thus, the incongruence between metastatic disease and the tasks of this developmental stage might have created pressure to display physical and psychological stamina all the time, explaining the participants’ insistence on staying strong.

Women in the present study also tended to stress the silver linings of the metastatic cancer experience, particularly through overemphasizing positive changes in their personalities. Inner strength has usually been emphasized as a protective factor enabling people to deal with cancer-related losses in a more adaptive way.^61,62^ It is also common for patients with cancer to think that their “after cancer” self is an improved version of them.^63–65^ This process has usually been described as “benefit finding” in patients with cancer^65–67^ and associated with enhanced quality of life.^61,68,69^ Accordingly, the emphasis of our participants on positive self-related changes might also be interpreted as a functional coping strategy. Despite physical difficulties, continuing their daily routines might be a way to pursue their lives as they were before the cancer diagnosis. Nevertheless, these findings need to be interpreted with caution. Between the lines, it was apparent that our participants’ minds were seesawed between psychological strength and physical limitations. Although benefit finding has also been observed among patients of different ages,^70,71^ young adults might feel more obligated to remain strong and therefore deny physical limitations. Under those conditions, remaining strong might result in lower quality of life since these individuals are not realistically prepared to face the difficulties associated with the process of metastatic illness.^67^

The second superordinate theme, “new ideals not on the agenda of a healthy young woman,” primarily refers to positive experiences and traits desired during the metastatic cancer process. Participants particularly expressed a need to develop a new personality to “beat cancer.” The characteristics of this new personality contrasted with the ones they had prior to diagnosis. Through attaining those ideal traits, they aimed to put cancer at bay. This finding partially supports patients with cancer urge to increase their sense of control over the disease process.^72–74^ The sense of control is particularly important in buffering negative psychosocial impacts of the disease and enhancing adaptive coping.^75,76^ Nevertheless, such an effort might be disappointing as it might lead to unrealistic expectations about treatment outcomes. The current literature does not provide robust evidence regarding the impact of personality on cancer development and recovery.^77^ Unfortunately, various genomic dynamics beyond the control of the patient seem to operate in the prognosis of the disease. Hence, mental health professionals should be cautious about such idealistic attitudes and help patients and their relatives develop cautious optimism. Young women with the metastatic disease also described normalcy as an ideal state to be attained. What they mean by “normalcy” is simply engaging in ordinary daily tasks without being constrained by disease-related difficulties. Interestingly, none of the participants mentioned any of the future projections that are typical for young adults (e.g., establishing a romantic relationship, having children, advancing in a profession).^38^ A possible explanation for lack of such projections might be related to our participants’ efforts to lower their expectations regarding their ideal selves. Since ideal-self traits would eventually be influenced by the realities brought by metastatic disease,^34^ they might try to reduce the tension between their actual and ideal selves by desiring only normalcy.

The third superordinate theme, “So-called ‘minimalist’ expectations from others,” pointed to the standards and attributes that young women felt obliged to possess after a metastatic cancer diagnosis. It seems that participants perceived recovery as an emotional debt to their parents. This finding might be closely related to the cultural context in which the study was conducted. Turkey is a Middle East country predominantly influenced by collectivist norms and values. In times of crisis, family members are mobilized and provide support to the affected individual to combat with the factors hampering group cohesion and harmony.^78^ Besides, expressing negative emotions has unfavorable cultural connotations since it is perceived as a threat to the group's wellbeing.^79^ Such cultural stance might explain the family members’ insistence on increasing the morale of young adults with metastatic cancer in the current study. Although such an encouraging attitude might be empowering to some extent,^80,81^ it might also increase the discrepancy between patients’ current situations and the attributes they are expected to possess. That is to say, the expectation that patients should never cry or feel sorry for themselves did not coincide with the reality of their current situations. In summary, when such expectations undermine the processing of losses and impairments, they might have a counteracting effect, increasing emotional burden and discomfort.^82,83^

Besides patients’ families, the healthcare staff also pressured the participants to control their negative feelings related to challenges in the prognosis and treatment of the disease. The therapeutic relationship between patients with cancer and healthcare workers is important when determining the quality of life and care satisfaction.^84–86^ However, due to the immediacy of the medical problems, healthcare professionals did not cater for patients’ psychosocial needs.^87^ Recent evidence has suggested that patients with advanced cancer might be particularly at risk of unmet emotional and social needs during inpatient and outpatient treatment.^88,89^ For example, although they were satisfied with the medical care provided by the health professionals, women with metastatic breast cancer reported poorer communication regarding quality-of-life issues.^90,91^ The findings of the current study also revealed a similar pattern. The participants felt emotionally unsupported as the medical team did not validate their need to express negative emotions. A possible explanation for this silencing might be related to the healthcare workers’ negative attitudes about metastatic cancer. Despite advances in early detection and treatment, cancer still invokes significant fear in the public, and healthcare providers are not free of having negative attitudes about prognosis and treatment.^92^ Thus, the severity of the disease might have led the medical team to avoid discussing the emotional toll.^93^ Besides, the cultural context might have created another barrier preventing medical staff from establishing an open dialogue about the patients’ emotional burden. Existing findings indicated that communication in cancer care varies depending on the cultural values adopted by patients and family members.^93^ For example, formal disclosure of the diagnosis is discouraged by family members in societies where group harmony and family unity are highly valued.^94^ Thereof, the medical team might have underestimated the emotional turmoil experienced by the participants, resulting in feelings of invalidation and loneliness. Lack of communication between young women and healthcare workers might have also intensified the patients’ emotional conflict regarding having advanced cancer at such a younger age.

## Limitations and directions for future studies

It is unfortunate that this study did not include more than one interview with each patient, but this limitation can be evaluated from two standpoints. First, it is an advantage to have more than one interview with the same participant to obtain detailed information in qualitative studies.^44^ However, since this sample required reaching patients with metastatic cancer, it was difficult to interview some participants due to their disease and treatment-related hardships. Another limitation of the present study is related to the sample characteristics. The study sample consisted of young adult women with metastatic cancer. Yet, the researchers were unable to homogenize the sample based on cancer and treatment types due to practical difficulties encountered during the data collection. The existing literature indicated that variability in cancer types and treatment modalities might impose differential impacts on the psychosocial wellbeing of the patients.^95,96^ Thus, future work should be conducted to investigate the unique psychosocial experiences of young adults diagnosed with particular metastatic cancers. Also, the current study was conducted with only female participants due to practical reasons described in the methods section. The participants consisted of only female young adults who were not in a romantic relationship at the time of the study and did not have children. It has been well-established in the literature that a cancer diagnosis might destabilize romantic relations in intimate and sexual aspects.^97,98^ Adults with younger children might have specific parental needs and concerns during the treatment process.^99^ As young adulthood years correspond to the reproductive ages, further work to understand the self-discrepancy experiences of women with romantic partners and children is needed.

## Clinical implications

The findings provided a detailed explanation of the self-conceptualizations of this group and the changes experienced throughout the disease process. Our study also reflects basic areas where young patients with metastasized cancer have difficulties, such as unrealistic expectations and necessities arising from their young age. Furthermore, it also provides information about the coping mechanisms that young women use in life-threatening situations. Accordingly, the current study provides a preliminary basis for intervention programs specifically designed for young women with metastatic cancer diagnoses. Existing intervention programs mainly focus on alleviating psychological symptoms such as anxiety and depression.^100^ However, understanding young patients’ priorities in the reorganization of self-perceptions seem to be an important issue throughout this process.

## Conclusion

The aim of this study was to investigate the self-concepts and discrepancies between the selves of young adult with metastatic cancer. There is no previous study in this context to the authors’ best knowledge. It has been seen that there are important changes in the person's actual, ideal, and ought selves. Essentially, there are difficulties due to the emphasis on being strong and conflicting emotions. While evaluating the wellbeing of these patients, it is important to pay attention to the desire to look strong. Besides, families and healthcare workers should encourage emotional expression to combat developmental and cultural barriers preventing the processing of cancer-related losses.

The findings of the current study suggest that young adult women might be frequently using denial throughout the disease process, and this overuse might be closely related to facing a life-threatening situation at a young age. In summary, being “stuck” in a young body while facing death seems to take a unique emotional toll on young adult patients with cancer.

## Appendix 1

### The intervıew questıons

1. Could you tell about your cancer diagnosis process? (How did you learn the diagnosis?)
  - How did you feel when you first heard about the diagnosis?
    - What did you do to deal with this feeling?
  - How did cancer diagnosis affect you/your life?
  - How did the treatment process affect you/your life?
2. How would you describe yourself as a person?
  - What are your main features?
    - How did you experience this feature in the disease process?
  - What are your main features according to those around you?
    - Who are the people you care about in your life? What are your main features according to them?
  - What are your expectations from yourself?
    - What is your most important role for you? What are your expectations from yourself according to that role?
    - Which of these do you think you met?
    - Which of these do you think you did not meet? Why is that?
  - What are the expectations of those around you from you?
  - What are the things you feel you are obligate to do?
  - Have there been any changes in these?
    - How do you cope?
    - Do you have any features that you are pleased to change?
    - Do you have features that you are not satisfied with the change?
3. What are your characteristics that help you in the diagnosis and treatment process?
  - What are your features that make the situation difficult for you?
  - What are the features that you think you do not currently have, but if you have them, what will help you?
